# The effect of a peer on VO_2_ and game choice in 6–10 year old children

**DOI:** 10.3389/fphys.2014.00202

**Published:** 2014-06-02

**Authors:** Lee A. Siegmund, Jonathan B. Naylor, Antonio S. Santo, Jacob E. Barkley

**Affiliations:** ^1^The Cleveland Clinic, Department of Preventive Cardiology and RehabilitationCleveland, OH, USA; ^2^College of Education, Health and Human Services, Kent State UniversityKent, OH, USA; ^3^Department of Exercise Science, University of Las VegasLas Vegas, NV, USA

**Keywords:** peer influence, motivation, video games, relative reinforcing value, physically interactive

## Abstract

Relative to sedentary video games (e.g., Playstation 2®), playing physically active video games (e.g., Nintendo Wii Sports Boxing®) significantly increases caloric expenditure in children. Studies have demonstrated that the presence of a peer increases physical activity in children. We sought to determine if children would expend more energy and find playing the “exergame” (Wii) more motivating than the sedentary video game (Playstation 2) when with a peer. Seventeen children (age 8.5 ± 0.4 years) rested, played the sedentary video game and “exergame” for 10 min each, in two conditions: one in which the children rested/played the games alone (alone condition) and another in which they played with a peer (peer condition). Oxygen consumption (VO_2_), and liking (visual analog scale) was assessed for each 10-min condition. After three 10-min resting/gaming conditions, motivation was assessed using a relative reinforcing value task in which children performed computer mouse presses to gain additional access for either the sedentary video game or “exergame.” VO_2_ was greater (*p* < 0.001) during “exergame” play (mean = 12.17 ± 4.1 ml·kg^−1^·min^−1^) vs. rest (mean = 5.14 ± 1.46 ml·kg^−1^·min^−1^) and the sedentary video game (mean = 5.83 ± 2.1 ml·kg^−1^·min^−1^). During the peer condition, there were no significant differences (*p* > 0.05) in VO_2_ relative to the alone condition. In an exploratory analysis boys exhibited a greater (*p* = 0.02) increase in VO_2_ from rest to “exergame” (Δ 9.0 ± 3.7 ml·kg^−1^·min^−1^), relative to girls (Δ 4.9 ± 2.9 ml·kg^−1^·min^−1^). Boys showed a significantly greater increase (*p* = 0.05) in VO_2_ from the resting condition to “exergame” in the presence of a peer (Δ 11.1 ± 5.3 ml·kg^−1^·min^−1^) vs. the alone condition (Δ 6.8 ± 3.1 ml·kg^−1^ ·min^−1^). Liking was significantly (*p* < 0.001) greater for “exergame” (7.7 ± 1.9 cm) and the sedentary video game (8.3 ± 1.3 cm) relative to rest (4.0 ± 2.8 cm). Motivation for “exergame” significantly decreased (*p* = 0.03) from alone (340.8 ± 106.8 presses) to the peer condition (147.8 ± 81.6 presses).

**Conclusion:** VO_2_ was greater during “exergame” play relative to the sedentary video game. The presence of a peer did not increase VO_2_ during “exergame” play. Surprisingly, the presence of a peer decreased children's motivation to play “exergame” vs. the sedentary video game.

## Introduction

The percentage of overweight children age 6–19 years in the United States increased to 16% by 2002, nearly triple what it was in 1980, and reached 16.9% by 2009 (Ogden and Flegal, 2010; Ogden et al., 2012). While poor dietary behaviors play an important role in the obesity epidemic, understanding the causal mechanisms behind the inadequate physical activity of today's children is also of great importance if we are to address obesity in children. Presently, children 10–16 years of age rarely take part in vigorous physical activity (Strauss et al., 2001). Children are becoming increasingly sedentary and this is directly affecting adiposity (Rennie et al., 2005). Multiple studies have identified an inverse relationship between caloric expenditure via physical activity and fat mass (Rennie et al., 2005).

While many factors contribute to the rise in childhood inactivity, one factor may be the increased access to highly-motivating sedentary behaviors (i.e., behaviors that are associated with sitting) such as television, video games and computers. These “square screen” activities have become so alluring and reinforcing (i.e., motivating) that they are directly contributing to the increase in children's sedentary behavior (Sisson et al., 2009). In fact, children are spending so much time in front of computers and televisions that they are spending less time sleeping, are skipping meals and are eating faster and poorer quality foods (Van den Bulck, 2004; Van den Bulck and Eggermont, 2006). In the U.S., 47% of children age 2–15 years, spend more than 2 h per day in sedentary leisure activity and may sit for as many as 10 h per day (Rennie et al., 2005; Sisson et al., 2009). This is an important concern, given that it has been shown that even in the presence of daily physical activity, people who spend a great deal of sedentary time each day are at greater risk for health consequences (Owen et al., 2010).

While increased “square screen” use appears to decrease physical activity, increasing the likelihood of overweight and obesity, physically-interactive video games (i.e., “exergames”) such as the Nintendo Wii® are becoming more popular and playing these games has been shown to increase energy expenditure above what is seen in traditional video game-play in children (Penko and Barkley, 2010). Playing certain “exergames” elicits caloric expenditures that would constitute light to moderate intensity physical activities in children with males exhibiting greater energy expenditure during “exergame” play than females (Maddison et al., 2007; Lanningham-Foster et al., 2009; Penko and Barkley, 2010). Therefore, if children would forego their traditional sedentary “square-screen” use and replace it with “exergames,” physical activity behavior may increase.

Playing “exergames” vs. traditional sedentary games may result in an increase in energy expenditure; however children may not always select a physically interactive game if traditional, sedentary games are an option (Williamson et al., 2012). Recent evidence suggests that the presence of a peer has a positive effect on physical activity behavior in children (Rittenhouse et al., 2011). Social influences have been examined in college students using virtual competitors. Snyder et al. (2012) found that a social facilitation effect resulted in greater exercise intensity in students when they competed against a live competitor vs. an avatar. Similarly, when children play “exergames,” they are competing against either the game itself (i.e., an avatar), or another live competitor. Children have been shown to initiate activity due to the influence of peers, either through co-participation, modeling, or encouragement (Jago et al., 2009; Salvy et al., 2009). Further, when children feel accepted by their peers they are more likely to be physically active (Coppinger et al., 2010). The reverse is true as well; children who report low support from peers report less physical activity (Hohepa et al., 2007). However, the previous experimental examinations of the effect of peer influence on physical activity behavior in children have utilized exercise equipment (e.g., cycle ergometers) or simulated recess periods as the only physical activity options. No studies that we are aware of have experimentally examined the effect of peer influence on children's motivation to play an “exergame” vs. a traditional, sedentary video game. If peer influence affects video game play in a manner similar to what has been demonstrated in these other settings (e.g., simulated recess), the presence of a peer may increase a child's motivation to choose to play a physically-interactive video game over a sedentary alternative. This may, in-turn, reduce sedentary behavior.

The purpose of this study was to examine the effect of peer influence (i.e., the presence of a friend) on children's motivation (relative reinforcing value) for an “exergame” [(Nintendo Wii Sports Boxing®), Wii] vs. a traditional sedentary video game alternative (Playstation II Ready to Rumble®). We hypothesized that children would demonstrate an increase in energy expenditure as assessed by oxygen consumption (VO_2_) in the “exergame” relative to the sedentary video game. Additionally, we hypothesized that children would expend a greater amount of energy while playing an “exergame” with a peer vs. playing the same game alone. We also anticipated that, relative to playing alone, the presence of a peer would increase children's motivation to play an “exergame,” vs. a traditional, sedentary video game. Finally, we hypothesized that children would report greater liking (i.e., enjoyment) for the “exergame” in the presence of a peer vs. the “alone” condition.

## Materials and methods

Participants (*N* = 17) included 15 Caucasian and two African American, 6–10 year old children (*n* = eight girls). Each participant identified one, same-sex friend (*N* = 17 total friends) to participate in the *peer* condition with them. The participant was allowed to bring a friend of his or her choice to take part in the peer condition. The only restrictions were that the friend needed to be in the same age group (6–10 years old), the same sex as the participant and healthy enough to be physically active along with the participant. Parents of all participants and parents of the friends completed a health questionnaire in order to rule out any contraindications to physical activity. No children who participated in the study had any known cardiovascular, pulmonary, orthopedic, metabolic, cognitive, neurological, muscular, or behavioral concerns or impairments.

All participants came to the laboratory for two visits; *alone* and with a *peer*, which was completed in a random order. During the initial visit, children were measured for height and weight and all study procedures were explained. Parents of participants and parents of the participants' friends completed an informed consent form and were given the opportunity to ask questions prior to signing. Assent for the participants and the friend's was obtained verbally. All procedures were approved via the university institutional review board.

During each visit, research staff demonstrated video game play and participants and friends practiced on the Nintendo Wii Sports Boxing® (“exergame”) and Playstation II Ready to Rumble® (the sedentary video game) boxing games for five minutes each. After familiarization with each game, participants completed the following 10-min gaming conditions: seated resting first, then playing the sedentary video game and the “exergame” in a random order. In order to avoid a fatigue effect, the children were divided into two groups. Group one (*N* = 9) played the sedentary video game first and group 2 (*N* = 8) played the “exergame” first. The sedentary video game is a system in which the participant controlled a character (i.e., boxer) on the screen with a hand-held remote. In the alone condition for the sedentary video game, the child's boxer fights another boxer controlled by the game software. The child was allowed to pick his or her own boxers. In order to be consistent, all children played the sedentary video game at the beginner level and were seated. None of the children who participated in the study had played the sedentary video boxing game prior to the study. In the *peer* condition, the participant and his or her friend each used the hand-held controls to “fight” one another's boxer while seated in front of the monitor.

This particular “exergame” has been used in previous studies and has been shown to elicit a VO_2_ similar to that of moderate intensity physical activity (approximately 10.5–21 ml·kg^−1^·min^−1^) (Graves et al., 2008; Penko and Barkley, 2010). Penko and Barkley (2010) also found that Wii boxing is significantly more reinforcing than a similar sedentary video game for the lean children (Penko and Barkley, 2010). All of the participants in the study were familiar with the Wii boxing game. While holding the Wii remotes children mimic punching motions which signal the avatars within the video game to move with the child's movement. Children boxed a computer-controlled boxer in the alone condition and his or her friend in the *peer* condition. This game was also programmed for a beginner. Per manufacturer's instruction, children were standing during the “exergame” condition.

During seated rest and each 10-min gaming condition oxygen consumption (VO_2_ ml·kg^−1^ ·min^−1^) was recorded via indirect calorimetry. After completing each condition, participants were asked to indicate how much they liked each 10-min gaming condition by making a mark on a 10 cm visual analog scale (VAS) anchored by “do not like it at all” on the left and “like it very much” on the right. Participants also reported their ratings of perceived exertion (RPE) at the conclusion of each 10-min gaming condition. RPE was assessed via the validated OMNI scale (Roemmich et al., 2006). After completing these three 10-min conditions, as a measure of motivation, participants completed a relative reinforcing value computer game to earn access to 11 additional minutes of the “exergame,” the sedentary video game or a combination of both games (e.g., “exergame” for 6 min, the sedentary video game for 5 min). The relative reinforcing value computer task is an operant button pressing task that requires children to perform work in the form of computer mouse presses to gain access to one activity (“exergame”) vs. another (the sedentary video game).

Procedures for the *alone* and *peer* conditions were identical except that during the *alone* condition participants completed all procedures alone. During the peer condition, the participant and their friend played both video games together. However, data (VO_2_, RPE, liking, motivation) from the friend was not recorded. Friends' data were not used because the relative reinforcing value task can only be played by one individual (participant) at a time. This is due to the fact that during the relative reinforcing value task participants earned additional access to the “exergame” and the sedentary video game in any pattern of their choosing. If a pair of children both played the relative reinforcing value task, they could potentially earn different amounts of access to the two games. This scenario was avoided by having only one child per pair complete the task and the time they earned for the “exergame” and the sedentary video game was then used for themselves and their friend.

VO_2_ for the participant was measured using a Parvo® metabolic cart and the child wore a Hans Rudolph 7600® mask. It was important that the participant and the friend had a similar experience during each 10-min condition. For this reason the friend also wore the same kind of mask, however it was not attached to the metabolic cart and no data was collected. Participants were compensated with a $20.00 gift card to a local store. Participants' friends received a $10.00 gift card.

### Anthropometrics

Height and weight was obtained by an American College of Sports Medicine Certified Health Fitness Specialist^SM^. Each variable was measured 3 times and the median score was recorded. Height was measured to the nearest 0.1 cm and weight was measured to the nearest 0.1 Kg with a balance beam scale/stadiometer (Health O Meter, Alsip, I). Body mass index (BMI) was calculated as follows: Weight in kg/height in m^2^.

### VO_2_

Oxygen consumption (VO_2_ ml·kg^−1^ ·min^−^1) was recorded during each of the 2 gaming conditions as well as at rest. Data was recorded via indirect calorimetry with a calibrated metabolic cart (Parvo Medics®) using Hans Rudolph 7600® masks. Means for VO_2_ were then converted to kilocalories (Kcal) per minute and metabolic equivalents (METS). One MET estimates rest and is approximately 3.5 ml·kg^−1^ ·min^−1^. METS were thus calculated by multiplying the VO_2_ by 3.5. VO_2_ values are in ml·kg^−1^ ·min^−1^. In order to convert this to Kcals, we multiplied the VO_2_ value by the participant's weight in kg. and divided by 1000 to obtain the number of liters expended per minute. This value was then multiplied times 5 since approximately 5 Kcals are burned for every liter of oxygen consumed. The Hans Rudolph 7600® Masks were used to collect expired gases (VO_2_ and CO_2_) during indirect calorimetry. The masks allowed the children in the *peer* condition to talk with one another while we monitored VO_2_. We felt the ability to communicate was necessary as the study was assessing the impact of peer influence on children's video game play. It would not be possible to speak using the mouthpiece and nose-clips.

### Liking

After the participants completed a 10-min condition (seated resting, sedentary video game, or physically interactive video game), they were asked how much they liked the activity using a Visual Analog Scale (VAS). The VAS consists of a 10 cm line. On one end of the scale was “like it very much” and the other end read “do not like it at all.” The children were asked to plot a pen mark on the line, indicating how much they liked or disliked the activity. The measure of liking was the distance from the left hash mark on the VAS to the child's pen mark. Liking, measured in this manner, has been shown to be predictive of actual physical and sedentary activity behavior (Roemmich et al., 2008).

### RPE

RPE data was collected at the mid-point of each 10 min condition using the validated OMNI Rating of Perceived Exertion Scale to determine how tired the child felt during activity (Roemmich et al., 2006). No RPE data was collected from the friend. The scale's use was explained with a standardized set of instructions. Perceived exertion for this study was defined as “How tired did your body feel while you were playing the game?”

### Relative reinforcing value

The relative reinforcing value computer task is an operant button pressing task that requires children to perform work in the form of computer mouse presses to gain access to one activity (“exergame”) vs. another (the sedentary video game). The participants had two computer screens available to them, one for the purpose of earning points for the “exergame” and the other for the sedentary video game. Each screen had three simple shapes and the participant was informed that he or she could press the computer mouse to change the shapes. This is similar to playing a slot machine. When all three shapes matched, the child earned one point, representing one minute of game play. He or she was able to earn points for a total of 11 min of game play and was allowed to earn points on either or both screens, meaning he or she was then allowed to earn all 11 min for one game or divide the 11 min between the two games. The first point earned was set for a fixed ratio (FR) of 1. This means that for one press of the computer mouse, the child earned one point. For the next point the FR then doubled, meaning the child had to press the mouse 2 times to get the shapes on the screen to match. For each subsequent point/minute earned, the participant had to press the computer mouse double the number of times he or she pressed the previous time in order to get all the shapes on the screen to match. If all 11 min were earned for one game the FR increased as follows: 1, 2, 4, 8, 16, 32, 64, 128, 256, 512, and 1024 presses of the mouse. The FR only increased for a given game when the child earned a point for that game.

The participant earned access to a video game based on how much work he or she was willing to do to earn a minute of activity for each gaming condition. The output maximum (O_max_) was the maximal amount of responding that the child performed to earn a single minute for a given activity (Feda et al., 2012). O_max_ for the “exergame” vs. the sedentary video game in each of the two social conditions was the measure of relative reinforcing value (i.e., motivation). Relative reinforcing value is a valid predictor of children's actual physical activity behavior (Epstein et al., 1999).

### Statistical analysis

All statistical analyses were performed utilizing the statistical package for the social sciences (SPSS, Version 17, Chicago, IL). Significance level for all calculations was set a priori at α ≤ 0.05.

Physical characteristics (age, height, weight, BMI) of the participants were compared between boys and girls using independent-samples *t*-tests. Two social condition (alone, peer) by three gaming conditions (rest, “exergame,” the sedentary video game) analyses of variance (ANOVAs) with repeated measures on both factors was performed to assess differences in VO_2_, liking and RPE. As an exploratory analysis on the effect of sex, a two sex (boys, girls) by two social condition (alone, peer) by three gaming conditions (rest, “exergame,” the sedentary video game) analyses of variance (ANOVAs) with repeated measures on the final two variables was performed to assess differences in VO_2_, liking and RPE.

A two social condition by two gaming condition (“exergame,” the sedentary video game) ANOVA with repeated measures on both factors was performed to assess differences in O_max_ (i.e., the measure of relative reinforcing value or motivation). *Post-hoc* tests on any significant effects from the aforementioned ANOVA models were performed utilizing Two-Way ANOVAs and *t*-tests with the Benjamini and Hochberg False Discovery Rate correction for multiple comparisons (Benjamini and Hochberg, 1995).

## Results

Participants' physical characteristics are shown in Table 1. There were no significant differences (*p* ≥ 0.43) between boys and girls for age, weight, height, BMI, or BMI percentile (BMI %).

**Table 1:** **Physical characteristics**.

	**Age(years)**	**Weight (kg)**	**Height (cm)**	**BMI (kg·m^−2^)**	**BMI (%)**
Boys	8.8 ± 0.5	31.4 ± 2.2	132.2 ± 2.9	17.7 ± 0.5	73 ± 5.5
Girls	8.2 ± 0.5	33.5 ± 4.0	131.8 ± 4.5	18.7 ± 1.3	70.5 ± 9.3
Total	8.5 ± 0.4	32.3 ± 2.2	132.1 ± 2.6	18.2 ± 0.7	71.8 ± 5.1

Data are means ± s.e.m.

The means for VO_2_, liking and RPE during each game and social condition are shown in Table 2. VO_2_ was converted into kilocalories (kcal) which is shown in Table 3.

**Table 2:** **Omnibus table of means for VO_2_, liking, and RPE**.

	**Alone**			**With Peer**		
	**Rest**	**Sedentary**	**Exergame**	**Rest**	**Sedentary**	**Exergame**
VO_2_(ml·kg^−1^·min^−1^)	5.2 ± 0.7	5.82 ± 0.7	11.3 ± 1.2	5.1 ± 0.6	5.9 ± 0.7	12.9 ± 1.1
Liking (cm.)	3.4 ± 0.7	8.1 ± 0.6	8.2 ± 0.5	4.5 ± 0.9	8.5 ± 0.4	7.1 ± 0.7
RPE (scale from 1–10)	1.7 ± 0.5	2.1 ± 0.5	3.0 ± 0.7	2.0 ± 0.5	1.9 ± 0.6	4.3 ± 0.6

Data are means ± s.e.m.

**Table 3:** **Kilocalories (kcal) per minute**.

	**Rest kcal/minute**	**Sedentary kcal/minute**	**Exergame kcal/minute**
Boys	0.73 ± 0.22	1.00 ± 0.33	2.1 ± 0.59
Girls	0.92 ± 0.4	0.86 ± 0.46	1.72 ± 0.78
All	0.82 ± 0.32	0.94 ± 0.39	1.93 ± 0.69

Data are means ± s.e.m.

### VO_2_

There was a significant main effect for gaming (*p* < 0.001). Paired-Samples *t*-test revealed that the main effect for gaming was due to greater (*p* < 0.001) VO_2_ when playing “exergame” (12.2 ± 4.1 ml·kg^−1^ ·min^−1^; 3.5 ± 0.26 METS) relative to the resting (5.1 ± 1.5 ml·kg^−1^ ·min^−1^; 1.46 ± 0.1 METS) and the sedentary video game conditions (5.8 ± 2.0 ml·kg^−1^ ·min^−1^; 1.65 ± 0.13 METS). Children increased caloric expenditure from 0.82 ± 0.32 kcal·min^−1^ at rest to 1.93 ± 0.69 kcal·min^−1^ while playing the “exergame.” There was no significant difference (*p* = 0.08) between VO_2_ in the resting condition and the sedentary video game. There were no additional significant main or interaction effects (*p* ≥ 0.23) for VO_2_.

### Exploratory analysis for VO_2_

**Figure 1:**
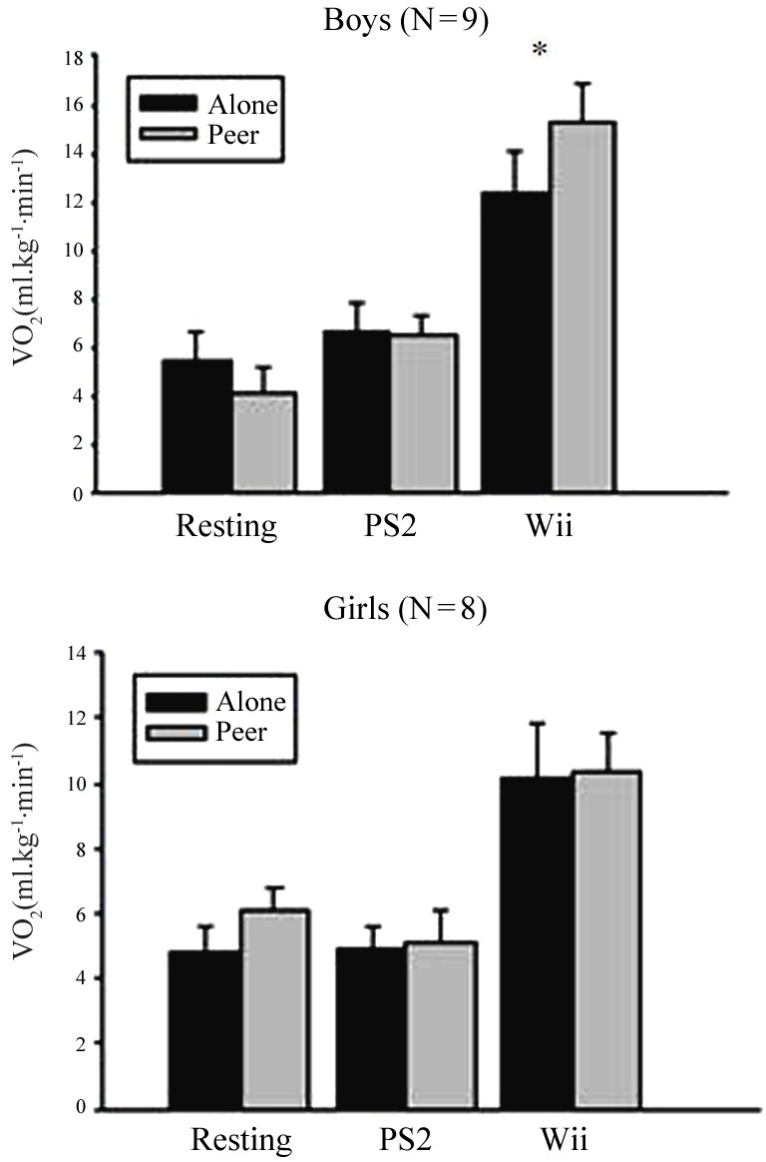
**VO_2_ for social condition by gaming condition by sex: mean ± s.e.m. for social condition by gaming condition by sex interaction for VO_2_**. In the peer condition males had significantly greater (*p* = 0.02) increase (Δ 11.1 ± 5.3 ml kg^−1^ min^−1^) in VO_2_ from rest to “exer game” (Wii™) versus the alone condition (Δ 6.8 ± 3.1 ml kg^−1^ min^−1^), (**upper panel**). Females showed no significant change (*p* ≥ 0.44) from alone to peer for any gaming conditions (**lower panel**). PS2, Playstation 2™ (sedentary game).

A significant social condition by gaming condition by sex interaction effect was found for VO_2_ (*p* = 0.04, Figure 1). To explore this Three-Way interaction, two additional social condition by gaming condition ANOVAs were performed for boys and girls separately. Boys had a significant social condition by gaming condition interaction (*p* = 0.02) whereas girls did not (*p* = 0.77). The interaction in boys was due to a significantly greater increase (*p* = 0.05) in VO_2_ from the resting condition to “exergame” in the presence of their peer (Δ 11.1 ± 5.3 ml·kg^−1^ ·min^−1^) vs. the alone condition (Δ 6.8 ± 3.1 ml·kg^−1^ ·min^−1^).

The increase in VO_2_ from the alone to peer conditions for boys in the sedentary video game vs. the “exergame” conditions (*p* ≥ 0.08) and in the rest vs. the sedentary video game conditions (*p* = 0.09) were trending toward significance. Girls showed no significant change in VO_2_ from the alone to the peer condition for any of the gaming conditions (*p* ≥ 0.44).

**Figure 2:**
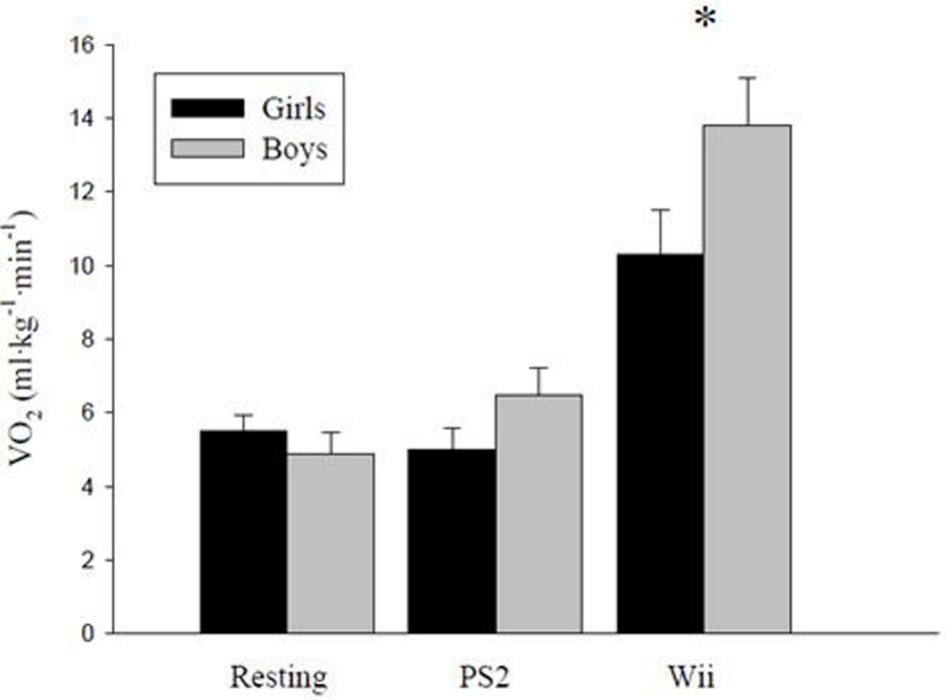
**VO_2_ for gaming condition by sex: mean ± s.e.m. for gaming condition by sex**. There was a significant increase (*p* = 0.02) in VO_2_ from the resting condition to “exergame” (Wii™) in boys but not girls. PS2, Playstation 2™ (sedentary game).

There was a significant gaming condition by sex interaction effect (*p* = 0.02, (Figure 2). This was caused by a greater (*p* = 0.02) increase in VO_2_ from the resting to the “exergame” condition in boys (Δ 9.0 ± 3.7 ml·kg^−1^ ·min^−1^) relative to girls (Δ 4.9 ± 2.9 ml·kg^−1^ ·min^−1^). Boys demonstrated an increase in caloric expenditure of 1.39 ± 0.61 kcal min^−1^ from the resting condition to the “exergame,” increasing from 0.73 ± 0.22 kcal·min^−1^ in the resting condition to 2.12 ± 0.59 kcal·min^−1^ in the “exergame” condition. Girls increased caloric expenditure 0.8 ± 0.45 kcal ·min^−1^ from the resting condition to “exergame,” increasing from 0.92 ± 0.4 kcal·min^−1^ at rest to 1.72 ± 0.78 kcal ·min^−1^ when playing the “exergame.”

### Liking

A significant main effect for gaming was found (*p* < 0.001). *Post-hoc* Paired-Samples *t*-tests revealed that this main effect was due to greater (*p* < 0.001) liking in the sedentary video game condition (8.3 ± 1.3 cm) relative to the resting condition (4.0 ± 2.8 cm) and greater (*p* = 0.001) liking in the “exergame” condition (7.7 ± 1.9 cm) relative to the resting condition. *Post-hoc* comparisons did not reveal any significant difference between liking for the sedentary video game vs. the “exergame” (*p* = 0.37). There were no additional significant (*p* ≥ 0.21) main or interaction effects for liking.

### RPE

There was a significant (*p* = 0.002) main effect for gaming condition for RPE. *Post-hoc* Paired-Samples *t*-tests revealed that this effect was due to a significant increase (*p* = 0.005) in RPE from the resting condition (1.9 ± 1.7) to the “exergame” (3.7 ± 2.3) and a significant increase (*p* = 0.014) from the sedentary video game (2.0 ± 1.6) to the “exergame.” There was no significant difference in RPE from the resting condition to the sedentary video game (*p* = 0.66). There were no additional significant (*p* ≥ 0.21) main or interaction effects for RPE.

### Relative reinforcing value (i.e., motivation)

**Figure 3:**
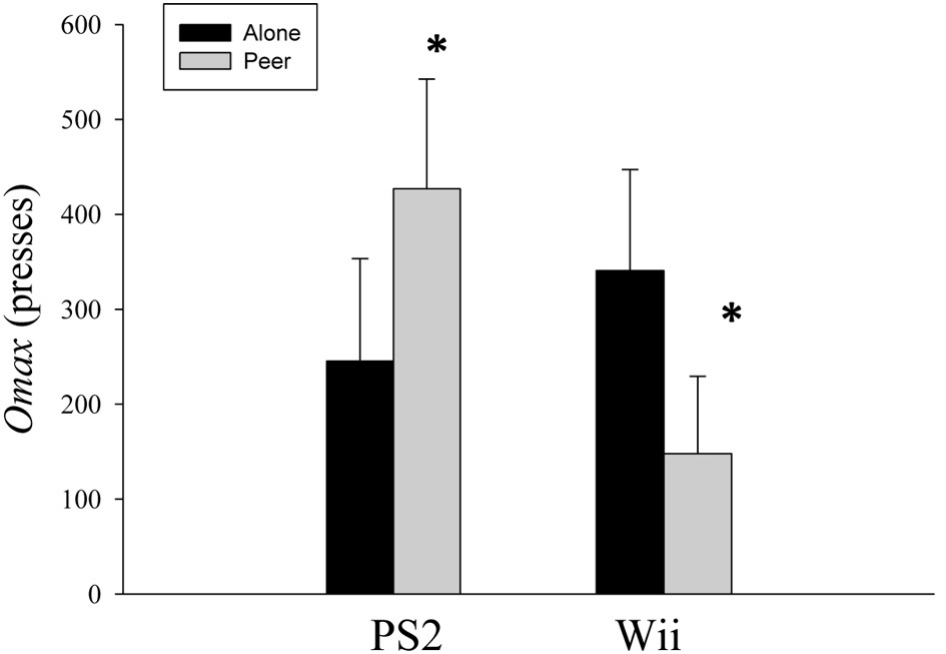
**Mean ± s.e.m. for O_max_ social by gaming conditions**. Sedentary video game significantly increased (*p* = 0.05) from the alone to the peer condition, while scores for the “exergame” significantly decreased (*p* = 0.04) from alone to the peer condition. ^*^Above the grey bar on the left = a significant (*p* = 0.05) increase in motivation for PS2 (sedentary game/Playstation 2™) from the alone condition to the peer condition. ^*^Above the grey bar on the right = a significant (*p* = 0.04) decrease in motivation for the Wii (exergame/Nintendo Wii™) from the alone condition to the peer condition.

There was a significant (*p* = 0.03) social condition by gaming condition interaction for O_max_ which was the measure of relative reinforcing value. *Post-hoc* Paired Samples *t*-Tests illustrated that O_max_ for the sedentary video game significantly increased (*p* = 0.05) from the alone condition (245.5 ± 108.0 presses) to the peer condition (427.0 ± 115.6 presses), while O_max_ for the “exergame” significantly decreased (*p* = 0.04) from the alone condition (340.8 ± 106.8 presses) to the peer condition (147.8 ± 81.6 presses, Figure 3). There were no additional significant main or interaction effects for O_max_ (*p* ≥ 0.25).

## Discussion

This is the first study we are aware of to examine the effect of the presence of a peer on children's metabolic expenditure and the motivation to play an “exergame” vs. a traditional sedentary video game (the sedentary video game). Surprisingly, children were less motivated (i.e., had a lower O_max_) to play the “exergame” in the presence of a peer relative to playing alone. This finding was in contrast to our hypothesis. As was the case in previous studies, energy expenditure and RPE was significantly greater during “exergame” play compared to the resting and the sedentary video game conditions (Barkley and Penko, 2009; Graf et al., 2009; Penko and Barkley, 2010).

Overall energy expenditure increased during exergame play, although there was no difference in VO_2_ between the two gaming conditions as a result of peer influence. Studies focusing on traditional physical activity behavior (e.g., playing in a gymnasium) have shown that children increase physical activity in the presence of a peer (Rittenhouse et al., 2011). This was especially true when the peer who is present is a friend as was the case in this present study. Previous research has also shown that exercise intensity increased when teenage and young adult women cycled against a live competitor vs. cycling alone or against a virtual competitor (Snyder et al., 2012). Unlike our study, social facilitation seems to have played a role in the cyclists' competitive nature, leading to increased energy expenditure. We found that not only did children fail to increase physical activity in the presence of a peer, they were less motivated to play the “exergame” when with a peer. These previous findings were the basis for our hypothesis that children would increase their physical activity with the “exergame” relative to a traditional sedentary alternative when playing with their friend vs. playing alone. While it is unclear why we found no peer effect for VO_2_, it would appear that the effect of peer influence on “exergame” is different than that of traditional physical activity. There may also be differences in the way children respond to competition relative to adults. Additionally, sex differences may have influenced some of our findings.

It is possible that there are differences in how males and females respond to gaming in social situations that affects energy expenditure. This may be especially true in a game that utilizes direct opposition, such as Wii boxing. In an exploratory analysis we found that VO_2_ did increase in the presence of a peer, but this was significant only when boys were examined separately. VO_2_ did not increase in the presence of a peer for girls. Sample size for boys and girls is small when examined separately (*n* = nine boys), but these findings provide intriguing results that may be useful for future investigations. This is similar to previous studies in both adults and children that have demonstrated greater average VO_2_ during “exergame” play in males vs. females (Graves et al., 2008; Barkley and Penko, 2009; Graf et al., 2009). Therefore it was not surprising that the boys, relative to girls, exhibited greater increase in energy expenditure during “exergame” play relative to resting. Since the current study utilized both a sedentary and a physically interactive boxing game, it is possible that the nature of the games was not as appealing to girls as to boys, thus affecting energy expenditure. Boys have been shown to demonstrate more peer directed physical aggression than girls (Maccoby and Jacklin, 1980). In our investigation the boys, relative to girls, exhibited greater VO_2_ during “exergame” play and the increase in VO_2_ from rest to “exergame” was also greater for boys in the peer condition. However, caution needs to be taken in the interpretation of these results since the sample size is small when analyzing sex as part of a 3-Way interaction. Therefore, we do not know if the sex differences in VO_2_ in the peer condition are spurious or real. It does lead to further questions about whether boys are truly more active in the presence of a peer and if certain gaming conditions affect this based on the more aggressive tendencies of boys. Further, we do not have enough evidence to postulate why the girls in our study were less active than boys when playing with a peer. Girls and young women between 17 and 22 years of age have demonstrated an increase in activity when competing against a peer in a non-aggressive “exergame” (Snyder et al., 2012). Future studies should examine whether girls may be more active in the presence of a peer under different (i.e., less aggressive) gaming conditions. While it is possible that girls do not like boxing games as well as boys, the children in our study did not like the “exergame” more than the sedentary game, either in the alone or peer conditions as we had hypothesized.

Motivation in our study was measured by obtaining an O_max_ for each gaming condition, using the relative reinforcing value task. The relative reinforcing value task revealed that O_max_ for the “exergame” vs. the sedentary video game was different across the two social conditions. Children exhibited a significant increase in O_max_ (i.e., work done to earn game time) to play the sedentary video game in the presence of a peer vs. alone, while at the same time significantly decreased O_max_ for the “exergame” when with a peer. Past research has demonstrated that “exergames” such as Wii are highly reinforcing, relative to a sedentary game, when children played the game alone (Penko and Barkley, 2010). We observed a similar effect in the present study as children exhibited a greater O_max_ for the “exergame” vs. the sedentary video game when alone. However, it was interesting and surprising that children's O_max_ for “exergame” declined when they were with a peer. There are a number of possible explanations for these unforeseen results.

Staiano et al. (2012) found that cooperative play resulted in greater intrinsic motivation to play the “exergame” than competitive play. Anecdotally, several of the children in our study asked if they could be on the same team rather than compete against each other. This raises the question as to whether the children felt that the “exergame” (Wii Boxing) was a first person experience, as though they were truly hitting or being hit by their friends. Wii Boxing requires the player to perform actual punching motions to play the game, which may make it more realistic than the sedentary video game. The sedentary video game may have felt less real to the children due to the cartoon-like graphics and the use of a controller as opposed to the more realistic movements (i.e., punches) the children were mimicking with the “exergame.” When the children played the sedentary video game they used a handheld controller in which pushing a button would cause the boxer on the screen to throw a punch. The boxers in the sedentary video game were various fantastical animated characters whereas the Wii boxer, although animated, was an avatar that actually represented the child's friend. It is possible that young children prefer not to be in direct opposition to their close friends and that the opposition associated with Wii Boxing was less appealing than the sedentary video game. Our study found that VO_2_ during direct peer opposition was not different from the alone condition and more interestingly, there was less motivation to be active when with a peer. The participants in Snyder et al. (2012), by contrast, may have found direct competition to be more motivating, resulting in higher energy expenditure in a similar peer condition. However, they were young adult women whereas we studied 6–10 year old boys and girls. It is also possible that young adult women are likely to increase activity while cycling against a peer, but may not do the same if they were boxing. The differences between our study participants as well as type of game relative to those of Snyder et al. (2012) may explain the dissimilar results.

An alternate explanation for our surprising findings may have simply been that children found it easier to converse with their friend when playing the sedentary video game vs. the “exergame.” We personally observed that the children were far more conversant with one another during the sedentary video game play. This is likely because the “exergame” required more physical exertion making conversation more difficult. Thus, the ability to interact with their peers may have been what fostered the greater motivation to play the sedentary game in the peer condition.

Many studies have demonstrated the ability of “exergames” to increase energy expenditure in children (Lanningham-Foster et al., 2006; Graves et al., 2008; Barkley and Penko, 2009; Siegel et al., 2009; Mitre et al., 2011; White et al., 2011) but unfortunately this does not always translate into the likelihood children will be motivated to play an “exergame” over a sedentary game. Our research illustrates that there can be a discordant relationship between the greater energy expenditure of an “exergame” and a lower motivation to play the game in the presence of a peer. The children in our study expended more than double the kilocalories playing the “exergame” than they did playing the sedentary alternative and yet motivation to play the “exergame” declined when the children were in the presence of a peer. Our results certainly present a challenge, but it may be that lack of intrinsic motivation in this case is not a barrier to providing greater opportunity for children to be physically active. Regardless, it can be said that while the presence of a peer did not increase VO_2_, energy expenditure increased with the “exergame” overall.

Motivating children to choose an “exergame” over a sedentary option is still a worthy inquest. Just as important however, may be examining ways to keep children active once they begin to play. The children in our study often expressed a desire to continue playing the “exergame” at the end of the 10 min gaming condition even though they later chose the sedentary game when offered a choice with the relative reinforcing value task. It has been shown that children will play an “exergame” 87% longer than traditional physically active games when in a free-choice gaming environment (Roemmich et al., 2012). This suggests that once they have begun to play an “exergame,” children will likely continue and expend more energy than they would if they remained sedentary. Thus, even if children opt for a sedentary game when given the choice, the “exergame” may be very motivating once they start playing it. The challenge then may be to simply initiate the activity in the first place.

Several limitations existed in the current research. First, our study was relatively small (*n* = 17), however previous studies have also shown an increase in energy expenditure with “exergames” using similar size or even smaller (Lanningham-Foster et al., 2006; Maddison et al., 2007). The differences in energy expenditure between boys and girls in the peer condition may not be valid due to the smaller sample sizes when sex is examined separately. However, the results are important to discuss as there may be a legitimate difference that should be examined in future research. Additionally, there was only the option of boxing for this particular study which may have limited motivation for all of the children and influenced liking, especially for the girls. Since girls have been shown to be less physically aggressive than boys, it is possible that girls found boxing to be too combative (Maccoby and Jacklin, 1980). Wii boxing was chosen due to the potential for greater energy expenditure compared to other games (Mitre et al., 2011; Roemmich et al., 2012). The game for the sedentary video game was also a boxing game, which allowed us to compare sedentary boxing to physically interactive boxing with two players. However, as mentioned previously, the fact that both games were competitive may also have limited liking and motivation for children who prefer cooperative play. It should also be noted that all of the children were familiar with and had played the Wii boxing game. At the same time, none of the children were familiar with the sedentary video game. It is possible that the novelty of the sedentary video game affected the motivation or liking of the games and is therefore an additional limitation of this study.

## Conclusion

The presence of a peer during the “exergame” did not increase energy expenditure relative to playing alone. When differences between boys and girls were examined, only boys were found to be more active in the peer condition. The presence of a peer resulted in decreased motivation to play exergames. It is possible that this decreased motivation was the result of a diminished ability to socialize with a peer during the “exergame” vs. sedentary video game play. It may also be that children perceived the “exergame” in the peer condition as more realistic than the sedentary video game and they did not want to be in direct opposition with their friends. For this reason, an examination of cooperative play vs. competitive play would be an important direction for further study. Since energy expenditure did increase with the “exergame” relative to the resting and the sedentary video game conditions, “exergames” remain a worthy area of ongoing and future research examining physical activity behavior in children.

## Funding

Funding for this research was provided by grant money from the Kent State University School of Health Sciences.

### Conflict of interest statement

The authors declare that the research was conducted in the absence of any commercial or financial relationships that could be construed as a potential conflict of interest.
